# Development of a SYBR Green-Based Real-Time PCR Assay to Detect *Oncomelania hupensis quadrasi* DNA in Environmental Water Samples

**DOI:** 10.3390/tropicalmed10050140

**Published:** 2025-05-20

**Authors:** Daria L. Manalo, Jude Karlo G. Bolivar, Karl Ian T. Ermino, Jeromir G. Bondoc, Mark Joseph M. Espino, Efraim P. Panganiban, Kathyleen S. Nogrado, Raffy Jay C. Fornillos, Mario A. Jiz, Lydia R. Leonardo, Ian Kendrich C. Fontanilla

**Affiliations:** 1 Department of Health, Research Institute for Tropical Medicine, 9002 Research Drive, Filinvest Corporate City, Alabang, Muntinlupa City 1781, Philippines; 2 Institute of Biology, University of the Philippines Diliman, Quezon City 1101, Philippines; 3 Department of Science and Technology, Science Education Institute, Taguig 1631, Philippines; 4 Department of Molecular Tropical Medicine and Genetics, Faculty of Tropical Medicine, Mahidol University, Bangkok 10400, Thailand; 5 Immunology Department, Research Institute of Tropical Medicine, Manila 1781, Philippines; 6 Office of Research Coordination, University of the East, 2219 C.M. Recto Avenue, Brgy. 404, Zone 41, Sampaloc, Manila 1008, Philippines

**Keywords:** schistosomiasis, *Oncomelania hupensis quadrasi*, environmental DNA, real-time PCR

## Abstract

*Oncomelania hupensis quadrasi* is the intermediate host of *S. japonicum*, the causative species of schistosomiasis in the Philippines. Conventionally, risk areas are identified by procedures requiring highly skilled personnel and constant surveillance efforts. Recent developments in disease diagnostics explore the utilization of environmental DNA as targets for polymerase chain reactions in disease surveillance. In this study, a low-cost, specific, and efficient SYBR Green-based real-time PCR assay to detect *O. h. quadrasi* DNA from water samples was developed, optimized, and validated. Primers were designed based on the A18 microsatellite region of *O. h. quadrasi.* The assay exhibited a detection limit of one copy number per microliter at 99.4% efficiency and R^2^ = 0.999, which specifically amplified *O. h. quadrasi* DNA only. Validation of this assay in environmental water samples demonstrated 100% PPV and NPV values, suggesting its potential as a tool for identifying risk areas, pathogen-directed surveillance, policy making, and disease control.

## 1. Introduction

Schistosomiasis japonica remains a significant public health threat in the Philippines, causing morbidities in most endemic rural communities and usually affecting school-aged children, farmers, and fishermen. This parasitic infection is considered both a water-borne and a snail-borne zoonosis. Despite a national schistosomiasis prevalence in the Philippines of less than 1%, the variation remains very high, with some areas in Northern Samar reporting a 48% prevalence of the disease [1,2] The presence of the snail intermediate host in water sources implies the potential for transmission of the disease to humans since the infective larval stage, called cercaria, develops inside the snail and is shed into surrounding freshwater [3]. This cercariae can, then, penetrate the skin of any mammal that may come in contact with the infected water [4]. In the Philippines, the pomatiopsid snail *Oncomelania hupensis quadrasi* serves as the intermediate host of *Schistosoma japonicum*. These snails can be found in both natural and man-made waterlogged and shady areas characterized by lush vegetation [5].

Effective monitoring and disease surveillance for schistosomiasis in the Philippines should be established alongside stringent control measures, such as case detection, chemotherapy (mass drug administration and selective treatment), health education, environmental sanitation, and snail control, to control the disease [6]. Most monitoring strategies to estimate the progress of disease intervention are performed through prevalence surveys in both human and other animal reservoir hosts using various diagnostic techniques, such as stool microscopy, stool concentration techniques, and serological tests [7]. Searching for and mapping snail habitats is labor-intensive due to the wide areas where snail sites are located and other factors such as highly varied landscapes. Aside from the problem of the inaccessibility of snail sites, conducting a snail survey poses hazards such as treading on slippery slopes or getting stuck in muddy substrates that can be deep. In some sites, the snail population density has dropped to a very low level, making the detection of snails extremely difficult [8].

Advances in molecular biology have been recently explored in disease diagnostics, in particular the use of polymerase chain reactions (PCRs). Guan et al. [9] illustrated the effects of significant genetic variations among different geographic populations on the distribution of *O. hupensis* using multiplex PCR in eight screened polymorphic microsatellite DNA loci. Ever since the microsatellite DNA were isolated from thousands of microorganisms, including mollusks, the library of *O. hupensis* microsatellite DNA has been the basis for the development of molecular methods targeting the DNA of *O. h. quadrasi* in water where they could thrive, or in monitoring areas which have previously been considered free of the parasite. These microsatellite DNA serve as important molecular markers since they are ubiquitous (occur throughout the eukaryotic genome) and have a high copy number. The development of a PCR-based detection assay for *O. h. quadrasi* in environmental samples like water provides results of high specificity and accuracy; hence, it is a potential parameter in the prediction and identification of snail habitats, as well as schistosomiasis in high-risk areas.

In the development of a qPCR assay, two methods can be utilized: SYBR Green and Taqman probes. SYBR Green is relatively cost-effective and easy to use and technically based on the binding of fluorescent dye to double-stranded deoxyribonucleic acid (dsDNA), whereas the TaqMan method is relatively more expensive and is based on dual-labeled oligonucleotide and exonuclease activity of the Taq polymerase enzyme [10,11].

Environmental DNA (eDNA) detection is a very useful tool to examine biota present in a sample, such as air, soil, and water, and for the detection of macroscopic and microscopic organisms [12,13,14,15]. The advantage of eDNA analysis is that it provides a fast and cost-effective survey of the presence of organisms of interest in an environmental sample. Environmental DNA analysis has been reported in many studies on the detection of pathogenic organisms. In the context of helminths, to date, the detection of trematodes *S. japonicum* in China [16] and *O. viverrini* in Lao PDR [15] in water samples has been successful. A study was conducted in the Philippines, wherein field-water samples from snail sites tested positive for both *S. japonicum* and *O. h. quadrasi* [2]. Calata et al. [17] show that, aside from water samples, soil samples can also be used in the detection of *O. h. quadrasi* eDNA.

The use of eDNA from water samples is, therefore, a safer and more reliable alternative in field surveys for schistosomiasis. This can minimize the potential risk associated with exposure to cercariae-contaminated water during malacological surveys. This study was conducted to develop and optimize a SYBR Green real-time PCR detection assay to detect the presence of *O. h. quadrasi* DNA in the collected water samples. This would eliminate the need for probe design and synthesis; hence, the cost-effectiveness of the assay. With the development of a qPCR assay for eDNA detection in freshwater samples, the effective monitoring of areas of significant transmission and the near elimination of the disease may be attained, which sometimes cannot be addressed through conventional methods.

## 2. Methods

### 2.1. Ethical Considerations

The biosafety clearance for utilization of this protocol was granted by the Research Institute for Tropical Medicine Biorisk Management Office (Muntinlupa City, Philippines). The gratuitous permit was acquired from the Bureau of Fisheries Region 2 (R02-0009-18). Institutional Animal Care and Use Committee certification was deemed unnecessary because there were no live vertebrates used to conduct this study.

### 2.2. Primer Design

An A18 microsatellite sequence (166 bp, accession number: GU204047.1) specific to *O. h. quadrasi* DNA served as the reference sequence to design the primers. Forward and reverse primers were then individually picked according to the recommended optimal primer conditions. Primer-BLAST (https://www.ncbi.nlm.nih.gov/tools/primer-blast/ (accessed on 27 March 2025)) [18] was used to initially verify the specificity of the primers chosen for the target, taking into consideration the number of hits (organisms aligning with the target) and the percent identity of hits or amplified targets. The chosen sequences were then sent out for sequencing (Integrated DNA Technologies, Inc., San Diego, CA, USA).

### 2.3. PCR Controls

Genomic DNA isolates from *O. h. quadrasi* snails were quantified using a DS-11 Series UV-Vis (DeNovix Inc., Wilmington, DE, USA) spectrophotometer, and then were used as positive controls (10^4^ DNA copy number); extracted artificial pond water (APW), on the other hand, was the negative control used.

### 2.4. Water Samples from Snail Aquaria

Wastewater from snail aquaria at the Immunology Department of the RITM was collected. Three collections were performed for each aquarium. For the negative control, artificial pond water was used. The water samples were then filtered.

### 2.5. Water Filtration and Sample Fixation

The water samples collected were filtered using a handheld portable pump (Nalgene, Thermo Fisher Scientific Inc., Waltham, MA, USA). Each water sample (500 mL) was filtered using 1 filter paper disc (GF/F, 0.7 μm; Whatman, Loughborough, UK). Before filtration, an equipment blank was made by filtering 500 mL of distilled water into the filtration device. Then, a batch of samples was subsequently run. One equipment blank corresponded to one batch of samples processed. This step was performed until all the samples were filtered. After filtration, fixation of the samples was performed by adding 70% ethanol, just enough to submerge the filter paper. The ethanol-fixed filter papers were individually packed in an aluminum foil sheet, labeled, and stored at −20 °C until further processing [2].

### 2.6. DNA Extraction and Quantification

Genomic DNA extraction was performed using a DNeasy^®^ Blood and Tissue Kit (QIAGEN, Hilden, Germany), with few modifications to the homogenization and lysis steps. The filter paper was cut into small pieces using sterilized scissors and inserted into a clean 2.0 mL microcentrifuge tube. Five hundred microliters of buffer AL and fifty microliters of Proteinase K were then added to the tube. The mixture was vortexed until completely mixed. The tube was then incubated at 56 °C for 30 min. After the incubation period, the rest of the extraction process was conducted following the manufacturer’s instructions. The extracted DNA was quantified using a DS-11 Series UV-Vis (DeNovix Inc., Wilmington, DE, USA) spectrophotometer. The elution buffer from the extraction kit was used to blank the equipment. All quantification readings in ng/µL were noted. Only samples with positive quantification readings were used for downstream processing.

### 2.7. Master Mix Preparation

A quantitative polymerase chain reaction (qPCR) was employed using a CFX96 Touch Real-Time PCR Detection System (BioRad Laboratories, Hercules, CA, USA). A PCR master mix was prepared using 10 µL SsoAdvanced™ Universal SYBR^®^ Green Supermix PCR kit (BioRad Laboratories, USA), 1 µL each of forward and reverse primer, and 5 µL of nuclease-free water. A total of 3 microliters of the extracted DNA was individually added as a template to make up a 20-µL-volume reaction.

### 2.8. Initial qPCR Conditions

The initial cycling profile used was a 95 °C activation step for 3 min, followed by 40 cycles of denaturation, annealing and extension, 95 °C (30 s) and 60 °C (15 s), respectively, followed by the instrument’s default melt curve setting. Annealing and extension times were adjusted based on the kit’s troubleshooting guide, particularly depending on the thermal cycler specifications and the size of the target.

### 2.9. Optimization of qPCR Reaction Conditions

Multiple parameters were considered and tested to obtain the optimal reaction conditions for the assay. These include primer concentration, primer annealing temperature, primer stability, assay specificity, assay limit of detection, and assay repeatability. The parameters were tested in triplicate using only positive controls and a no-template control. Corresponding Ct values, mean, and standard deviation were noted accordingly.

#### 2.9.1. Annealing Temperature

The optimal annealing temperature was determined using a temperature gradient ranging between 56 °C and 65 °C. Specific temperatures within the gradient were pre-determined by an CFX96 PCR thermocycler (56.0 °C, 56.6 °C, 57.8 °C, 59.6 °C, 61.7 °C, 63.0 °C, 64.5 °C, and 65.0 °C). The optimal temperature was determined by the temperature that exhibited the lowest Ct value without non-specific amplification and with the lowest standard deviation.

#### 2.9.2. Optimal Primer Concentrations

A primer concentration gradient of various dilutions (10 µM, 5 µM, and 2 µM) of the stock primers (100 µM) was used to determine the optimal primer concentration for the assay under development. The lowest working concentration without non-specific amplification and with the lowest standard deviation was designated as the optimal primer concentration [19].

#### 2.9.3. Primer Stability

After determining the optimal primer concentration, primer stability was tested by running one positive control against twenty no-template controls (NTCs) using the optimized annealing temperature.

### 2.10. Assay Specificity, Sensitivity, and Efficiency

The optimized assay conditions where amplification was optimal were a 5 µM primer concentration with a 63 °C annealing temperature. The optimized PCR reaction conditions are 95 °C for 3 min of enzyme activation, 40 cycles of template denaturation, annealing, and extension at 95 °C for 30 s and 63 °C for 25 s, respectively, followed by a melt curve analysis. These conditions were employed in all downstream processes to further evaluate the assay’s laboratory performance, i.e., specificity to the target DNA and detection sensitivity, as well as its efficiency.

#### 2.10.1. Assay Specificity

Aside from a previously performed BLAST [20] search, laboratory specificity of the assay was also determined. This was achieved by evaluating the ability of the assay to distinguish between positive and negative samples. Moreover, DNA extracts from other snails that might co-habitate with *O. h. quadrasi* were used as negative controls. The following snails used: *Jagora asperata*, *Vivipara zamboagensis surigensis*, *Radix quadrasi*, and *Pomacea canaliculata*. All samples were run in triplicate.

#### 2.10.2. Assay Sensitivity

The sensitivity of the assay was determined by its limit of detection (LOD). A serial dilution (10^6^, 10^5^, 10^4^, 10^3^, 10^2^, 10^1^, 10^0^, 10^−1^, and 10^−2^ copies/µL) of the positive control of a known DNA copy number was tested with eight replicates each. The lowest dilution with a detection rate of 8/8 was determined to be the limit of detection.

#### 2.10.3. Assay Efficiency

A standard curve using dilutions of the positive control was established to determine assay efficiency. The dilutions were based on the LOD previously determined and were used as templates. One PCR reaction mix for each template dilution was prepared and tested using the optimized thermocycling conditions. PCR characteristics, such as percent efficiency, correlation coefficient (R^2^), and slope of the linear regression line, were also obtained from the standard curve.

### 2.11. Assay Validation Using Environmental Samples

The diagnostic specificity and sensitivity of the optimized assay were validated using thirty *O. h. quadrasi*-infected water samples collected from laboratory-maintained snail aquaria at the Immunology Department of RITM as positive samples. On the other hand, thirty *O. h. quadrasi*-negative samples were collected from freshwater bodies along known non-endemic areas in the Philippines, Muntinlupa City and Los Baños, Laguna. DNA extraction and PCR methods were employed as described above.

### 2.12. Assay Diagnostic Performance

Positive Predictive Value (PPV) and Negative Predictive Value (NPV) were calculated to validate the assay’s diagnostic performance. The PPV was calculated as the proportion of true positives correctly detected as positive by the method under evaluation, while the NPV was calculated as the proportion of true negatives correctly detected as negative.

### 2.13. Study Design Limitation

The assay was validated using a small sample size for both endemic and non-endemic areas, which may not accurately represent the population accurately.

## 3. Results

### 3.1. Primers

Primers were designed with reference to the A18 microsatellite sequence (166 bp, accession number: GU204047.1). Primer-BLAST results showed specificity to a single hit with 100% identity to a 184 bp product length of *Oncomelania quadrasi* DNA (accession number: LC276227.1). Forward and reverse primer sequences are presented in Table 1.

### 3.2. Optimization of qPCR Reaction Conditions

Assay development started by optimizing the primer annealing temperature (Table 2) and working concentrations (Table 3). Optimized conditions were determined by identifying the setup with the lowest Ct value, with no contamination in all replicates, and with the lowest standard deviation value [19].

**Figure 1:**
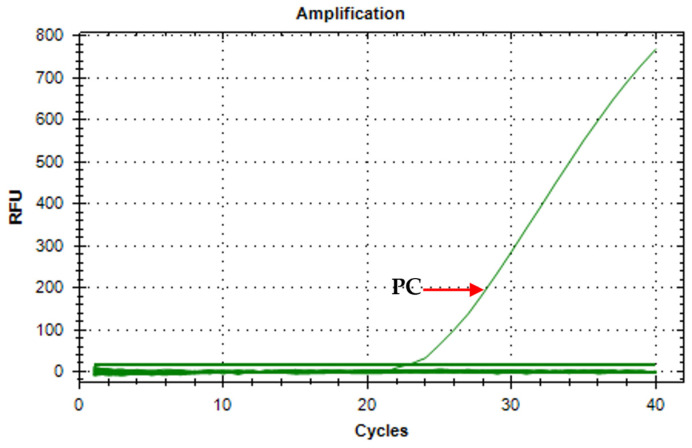
Amplification curve of A18 primers on *O. h. quadrasi* DNA against 20 no-template controls (PC = positive control).

Primer stability was tested to demonstrate that there is no primer–dimer formation in large amounts of the master mix preparation. One *O. h. quadrasi* DNA sample was run against twenty no-template controls (Figure 1). Results only show amplification in the *O. h. quadrasi* DNA (Ct = 22.57) and no amplification in all the no-template controls; hence, a stable primer set.

### 3.3. Assay Specificity

**Figure 2:**
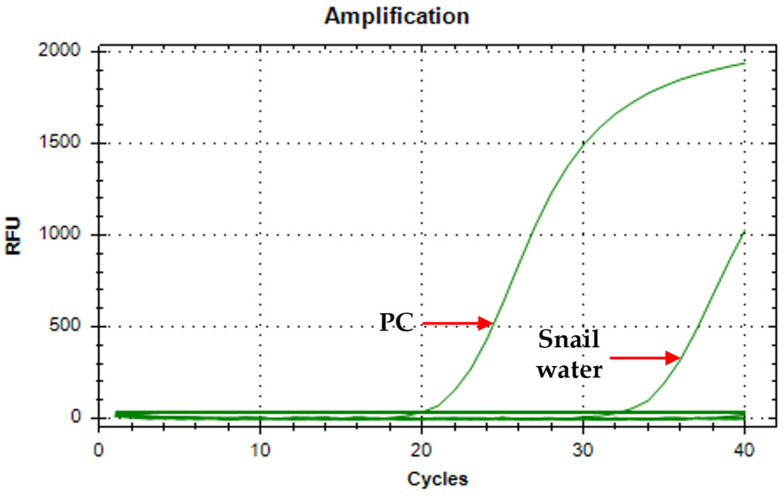
Amplification curve of A18 primers on *O. h. quadrasi* DNA and snail water (PC = positive control; snail water = water from aquaria).

The specificity of the assay was determined by initially running the extracted water samples from the snail aquaria. Artificial pond water was used as a negative control. The amplification curve (Figure 2) shows that only the positive control (Ct = 19.67) and the water from the snail aquaria (Ct = 32.02) tested positive in the assay. Both the negative control and the no-template control showed no amplification; hence, a valid run.

The assay was also used to distinguish *O. h. quadrasi* DNA from other snails that might co-exist in the natural environment (Table 4). The *O. h. quadrasi* DNA was run with the DNA of *J. asperata*, *V. z. surigensis*, *R. quadrasi*, and *P. canaliculata*; amplification was observed in the *O. h. quadrasi* DNA only (three out of three). Non-amplification in non-*Oncomelania* snails and artificial pond water with no snails present further validates assay specificity.

### 3.4. Assay Limit of Detection (LOD)

Dilutions of the positive control were used as templates and were replicated eight times. Table 5 shows the limit of detection with the lowest dilution at 8/8 (100%) amplification. The assay LOD is determined as 10^0^ copies/µL or 1 DNA copy number.

**Figure 3:**
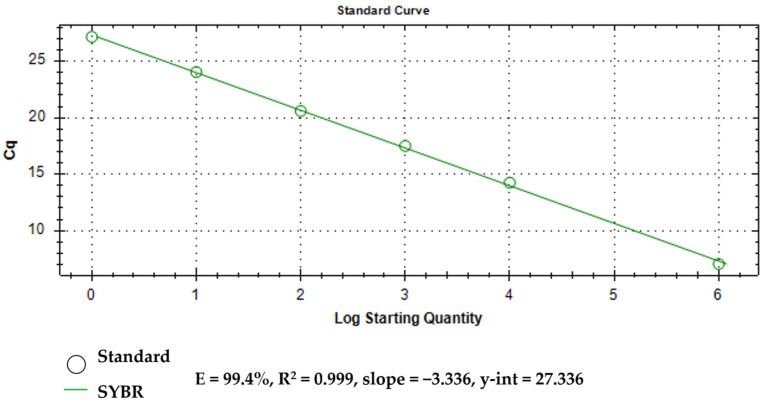
Standard curve of the *O. h. quadrasi* qPCR assay.

An established PCR standard curve demonstrates the efficiency of the assay. The assay obtained a standard curve slope of −3.336 with 99.4% efficiency and coefficient of correlation (R^2^) of 0.999 (Figure 3).

### 3.5. Assay Verification Using Environmental Waters

Verification of the assay’s diagnostic performance was conducted by using environmental water from the snail aquaria (positive samples) and water from non-endemic areas (where *O. h. quadrasi* are not found, i.e., negative samples). Thirty positive water samples from *O. h. quadrasi* snail aquaria and thirty negative water samples from freshwater bodies in non-endemic areas were used to evaluate diagnostic performance. The extracted water samples used in the validation process were initially quantified using a DeNovix spectrophotometer. This would validate successful DNA extraction from the water samples. Only those with positive spectrophotometric readings were used as PCR templates. A negative extraction control (NEC) was also included to detect possible contamination during the extraction process. No amplification was observed in the NTC and NEC, validating the PCR run.

Table 6 illustrates the diagnostic accuracy of the assay in detecting true positives as positive and true negatives as negative. A 100% PPV and 100% NPV value and acceptable PPV and NPV values at a 95% confidence interval were demonstrated in the assay (Table 7).

**Figure 4:**
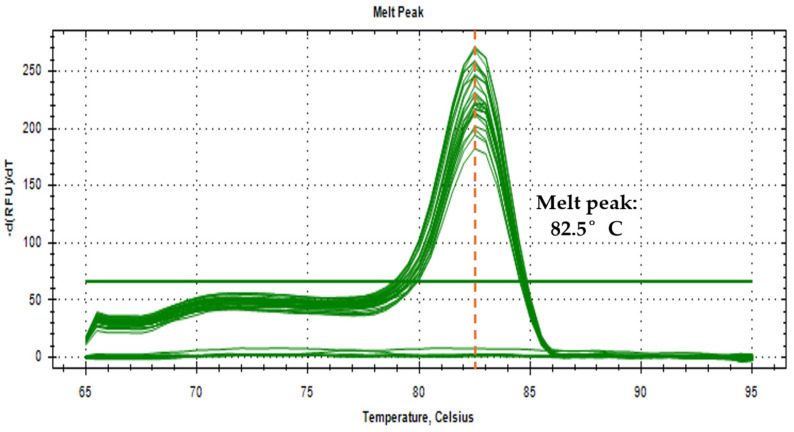
Melt curve analysis of amplified *O. h. quadrasi* PCR products with a melt temperature of 82.5 °C.

To further validate the correctness of the detection in environmental water samples in an SYBR Green detection platform, a melt curve analysis was employed. Similar melt peaks imply the similarity of the amplified target [22]. Figure 4 shows a uniform 82.5 °C melt peak for all samples, demonstrating specificity and accuracy in the detection of the target in the water samples.

## 4. Discussion

Schistosomiasis remains a major public health burden in the Philippines, partly due to the zoonotic nature of the disease and the prevalence of the parasite’s unique intermediate host, *Oncomelania hupensis quadrasi* [23,24]. To control the intermediate host is to disrupt the life cycle of *S. japonicum* and thus impede the parasite’s survival. The main objective of this study is to develop, optimize, and validate a real-time PCR detection assay for *O. h. quadrasi* eDNA in water samples.

Primer design and optimization are some of the most critical steps to attain a successful PCR assay. Several criteria described in the literature [25], such as length (bp), primer melting temperature (Tm), and %GC content, were considered in the design and choice of an appropriate primer sequence to be synthesized and utilized.

The forward primer has a length of 20 bp, while the reverse primer has a length of 21 bp. The ideal primer length is 18–24 bp to maximize primer–template hybridization in a highly specific manner. Designing a longer primer may result in slower hybridization, which can reduce specificity and efficiency, resulting in inadequate binding to the target sequence [26,27]. On the other hand, shorter primers may produce inaccurate, non-specific DNA amplification [28].

The melting temperature (Tm) is the temperature at which the DNA duplex is denatured into single strands. The primers used in this assay have almost similar Tm at 60.18 °C (forward) and 59.79 °C (reverse) to efficiently amplify a DNA sequence by synchronous molecular binding.

The oligonucleotides designed have a %GC content of 55% in the forward primer and 52% in the reverse primer. Stable binding of primers to templates is mostly achieved when primers contain 40–60% GC content. GC bonds contribute more to the stability (increased melting temperatures) of primer/template binding than of AT bonds. Higher GC content, however, can cause a mismatch and the formation of primer–dimers [29,30], resulting in low assay specificity.

The optimal annealing temperature (Ta) of this assay is 63 °C (Table 2), determined through a temperature gradient in the PCR cycling condition. The temperature range tested was between 56 °C and 65 °C. As a general rule, the annealing temperature should be set at 3–5 °C lower than the lowest Tm of the primers. The PCR kit used initially suggested an annealing temperature of 60 °C; however, during the optimization process, a more specific amplification was exhibited at 63 °C. A higher temperature improved the specificity by boosting discrimination against incorrectly annealed primers and increased the stringency of primer annealing. Furthermore, it minimized the misincorporation of incorrect nucleotides at the 3′ end of the primers. Increasing the annealing temperature, however, would result in a relatively lower yield, thus increasing annealing time from 15 s to 30 s [31].

A concentration gradient was employed to determine the optimal primer concentration of the assay. From a 100 µM stock primer concentration, dilutions were made at 10 µM, 5 µM, and 2 µM. Taking into consideration Ct value, mean, and standard deviation, a primer concentration of 5 µM (mean Ct = 19.68 and SD = 0.130) is optimal. All tests showed no amplification in the no-template control, validating the accuracy and correctness of the PCR process.

After establishing optimal PCR conditions, the assay’s laboratory performance was further validated in terms of specificity, sensitivity, and efficiency. The assay’s analytical specificity pertains to its ability to identify a negative result in samples without the target analyte [32]. The qPCR assay developed in this study specifically detects *O. h. quadrasi* DNA extracted from *O. h. quadrasi* snails (positive control) and snail aquaria wastewater.

Furthermore, the sensitivity of an assay is its ability to detect a positive result in a sample containing the target analyte [32]. This was demonstrated by determining the limit of detection (LOD). A lower LOD indicates a more sensitive assay [33]. For the qPCR, LOD can indicate the minimum concentration of nucleic acid that can be detected. An assay can detect up to one copy number per microliter. The qPCR should always yield a positive result when samples are at least at this concentration [34].

A PCR standard curve plots threshold cycle (Ct) values against the logarithm of known input amounts of a standard material to quantify the amount of target nucleic acid present in a sample [35]. Generally, a slope of −3.322 means that the PCR has an efficiency of 1, or 100%, and that the amount of PCR product doubles during each cycle. A slope of less than −3.322, as observed in this assay, indicates a PCR efficiency less than 1. Generally, most amplification reactions do not reach 100% efficiency due to experimental limitations. A slope greater than −3.322, on the other hand, indicates a PCR efficiency that appears to be greater than 100%, suggesting the presence of inhibitors in the reaction. The R^2^ value generally demonstrates that a specific value fits an observed trend [36], which can further imply the efficiency of the assay regardless of the amount of target analyte present in a sample.

The accuracy of these findings may further be validated by testing the assay with larger sample sizes from both endemic and non-endemic areas.

## 5. Conclusions

These findings further validate the assay’s capability as a diagnostic tool to detect the presence of *Oncomelania hupensis quadrasi* snails in a body of water which, in return, would suggest possible *Schistosoma japonicum* infection. It is then recommended that the assay be further validated using a larger sample size from *S. japonicum*-endemic areas. With the development of this qPCR assay for eDNA detection in freshwater samples and by further improving and validating its detection capability, effective monitoring of areas with significant transmission of the disease can be achieved. Furthermore, near elimination of the disease, if not its full eradication, may be attained. In summary, the utilization of this qPCR assay will play a vital role in the identification of risk areas and pathogen-directed surveillance, policy making, and disease control.

## Figures and Tables

**Table 1:** Forward and reverse *Oncomelania hupensis quadrasi* A18 primers.

Primer	Sequence	Length (bp)	Tm (°C)	%GC
Forward	5′-CCAGCGCAAAGCTCGTTTAG-3′	20	60.18	55
Reverse	5′-TCAAGAGAATCTCCAGGCACG-3′	21	59.79	52

**Table 2:** Determination of optimum annealing temperature (Ta) of A18 primers for *Oncomelania hupensis quadrasi* (Ohq) DNA using a temperature gradient (positive control at 10^4^ copies/µL).

Annealing Temperature (°C)	No-Template Control	Ohq Positive Control (Ct Values)				
		Replicate 1	Replicate 2	Replicate 3	Mean	Standard Deviation
56.0	Invalid	20.59	21.11	21.07	20.92	0.289
56.6	Invalid	20.53	20.96	21.22	20.90	0.348
57.8	Valid	20.61	20.74	21.6	20.98	0.538
59.6	Valid	19.51	19.06	20.11	19.56	0.527
61.7	Valid	19.63	19.83	19.65	19.70	0.110
63.0	Valid	19.51	19.53	19.47	19.50	0.031
64.5	Valid	19.67	19.65	19.83	19.72	0.099
65.0	Valid	19.51	19.62	19.61	19.58	0.061

**Table 3:** Determination of optimal concentration of A18 primers on *Oncomelania hupensis quadrasi* (Ohq) DNA using a concentration gradient (positive control at 10^4^ copies/µL and annealing temperature = 63 °C).

Primer Concentration (µM)	No-Template Control	Ohq Positive Control (Ct Values)				
		Replicate 1	Replicate 2	Replicate 3	Mean	Standard Deviation
1.0	Valid	31.11	30.09	30.26	30.49	0.546
2.0	Valid	21.49	21.78	21.39	21.55	0.202
5.0	Valid	19.81	19.67	19.55	19.68	0.130
10.0	Valid	19.52	19.27	19.02	19.27	0.250

**Table 4:** Determination of assay specificity using DNA from co-existing snails.

Snail Species	Ct Value		
	Replicate 1	Replicate 2	Replicate 3
*Oncomelania hupensis quadrasi*	19.37	19.44	19.41
*Jagora asperata*	NA	NA	NA
*Vivipara zamboagensis surigensis*	NA	NA	NA
*Radix quadrasi*	NA	NA	NA
*Pomacea canaliculata*	NA	NA	NA
No-template control	Valid	Valid	Valid

**Table 5:** Replicate testing to determine the limit of detection of the qPCR assay. NA: No Amplification.

Mean Concentration (Copies/µL)	% Replicate Detection N = 8	Mean Ct	Ct SD (≥95% CI)
10^6^	100 (8/8)	7.14	0.18
10^5^	100 (8/8)	14.57	0.50
10^4^	100 (8/8)	17.63	0.15
10^3^	100 (8/8)	21.03	0.18
10^2^	100 (8/8)	24.21	0.11
10^1^	100 (8/8)	26.84	0.30
10^0^	100 (8/8)	28.78	1.01
10^−1^	100 (7/8)	30.79	1.34
10^−2^	100 (7/8)	31.47	1.39
No-template control	0 (0/8)	NA	NA

**Table 6:** The results of the diagnostic accuracy evaluation of the Ohq qPCR assay using environmental samples.

		Ohq-Infected Environmental Water Sample	
		Positive	Negative
Ohq qPCR assay detection	Positive	30	0
	Negative	0	30

**Table 7:** The diagnostic performance (PPV and NPV) of the Ohq qPCR assay using environmental samples.

Performance Criteria	Estimated Value (%)	95% CI (%)
Positive Predictive Value (PPV)	100	88.43 to 100.00
Negative Predictive Value (NPV)	100	88.43 to 100.00
Computed using: https://www.medcalc.org/calc/diagnostic_test.php (accessed on 27 March 2025) [21]		

## Data Availability

The datasets used and analyzed during the current study are available from the corresponding author upon reasonable request.
